# 

**Published:** 1898-06

**Authors:** 


					﻿Southern Medical Record
A Monthly Journal of Medicine and Surgery.
Vol. XXVIII.	ATLANTA, GA., JUNE, 1898.	-;	, No. 6
BERNARD WOLFF, M. D;*	I
Associate Editors :
A. W. GRIGGS. M. D.
j. McFadden gaston. m. d
WILLIS F. WESTMORELAND, M. D
B. M. ZETTLER, Business Manager.
				

